# Influenza and SARS-Coronavirus Activating Proteases TMPRSS2 and HAT Are Expressed at Multiple Sites in Human Respiratory and Gastrointestinal Tracts

**DOI:** 10.1371/journal.pone.0035876

**Published:** 2012-04-30

**Authors:** Stephanie Bertram, Adeline Heurich, Hayley Lavender, Stefanie Gierer, Simon Danisch, Paula Perin, Jared M. Lucas, Peter S. Nelson, Stefan Pöhlmann, Elizabeth J. Soilleux

**Affiliations:** 1 German Primate Center, Göttingen, Germany; 2 Oxfabs, Nuffield Department of Clinical Laboratory Sciences, John Radcliffe Hospital, University of Oxford, Oxford, United Kingdom; 3 Institute of Virology, Hannover Medical School, Hannover, Germany; 4 Division of Human Biology, Fred Hutchinson Cancer Research Center, Seattle, Washington, United States of America; 5 Department of Cellular Pathology and Nuffield Department of Clinical Laboratory Sciences, University of Oxford, John Radcliffe Hospital, Oxford, United Kingdom; Kantonal Hospital St. Gallen, Switzerland

## Abstract

The type II transmembrane serine proteases TMPRSS2 and HAT activate influenza viruses and the SARS-coronavirus (TMPRSS2) in cell culture and may play an important role in viral spread and pathogenesis in the infected host. However, it is at present largely unclear to what extent these proteases are expressed in viral target cells in human tissues. Here, we show that both HAT and TMPRSS2 are coexpressed with 2,6-linked sialic acids, the major receptor determinant of human influenza viruses, throughout the human respiratory tract. Similarly, coexpression of ACE2, the SARS-coronavirus receptor, and TMPRSS2 was frequently found in the upper and lower aerodigestive tract, with the exception of the vocal folds, epiglottis and trachea. Finally, activation of influenza virus was conserved between human, avian and porcine TMPRSS2, suggesting that this protease might activate influenza virus in reservoir-, intermediate- and human hosts. In sum, our results show that TMPRSS2 and HAT are expressed by important influenza and SARS-coronavirus target cells and could thus support viral spread in the human host.

## Introduction

Influenza viruses and the SARS-coronavirus (SARS-CoV) are highly transmissible respiratory viruses which pose a serious threat to human health. The yearly recurring influenza epidemics are associated with significant morbidity and mortality, particularly among the elderly, and the global spread of pandemic influenza viruses can cause millions of deaths [1]. The severe acute respiratory syndrome coronavirus (SARS-CoV), which causes a novel lung disease, SARS, emerged in 2002 and spread to 26 countries in 2003, with 774 fatal infections [2]. Both SARS-CoV and influenza viruses circulate in animal reservoirs, water fowl (influenza) and bats (SARS-CoV) [3], [4]. Therefore, the identification of cellular factors essential for viral spread in animal and human cells should allow novel approaches to prevention and therapy.

The SARS-CoV spike protein (SARS-S) and the influenza virus hemagglutinin (HA) are inserted into the viral membranes and mediate host cell entry. For this, SARS-S and influenza HA bind to host cell receptors, ACE2 (SARS-CoV) [5] and 2,6-linked sialic acid on membrane proteins or lipids (human influenza viruses) [6], and mediate the fusion of the viral membrane with a host cell membrane. As a consequence, viral components are released into the host cell and can subvert the synthetic capabilities of the host cell for production and release of progeny particles.

The influenza HA and the SARS-S-protein are both synthesized as inactive precursors which transit into their active forms upon cleavage by host cell proteases. Cleavage of SARS-S and influenza HA is essential for viral infectivity and the responsible proteases are targets for antiviral intervention [7], [8], but their nature is incompletely defined. Recent evidence indicates that the type II transmembrane serine proteases (TTSPs) TMPRSS2, TMPRSS4 and HAT can activate human influenza viruses for spread in protease transfected cells [4], [9], [10]. In addition, endogenous TMPRSS2 was shown to promote influenza virus spread in the cell lines Caco-2 and Calu-3 [11], [12]. The SARS-CoV was found to be activated by cathepsin L upon viral uptake into host cell endosomes [8]. However, several recent reports demonstrated that expression of TMPRSS2 in target cells rendered cathepsin activity dispensable for infectious entry of SARS-CoV [13]–[15], suggesting that both SARS-CoV and influenza viruses can exploit TTSPs to promote their spread.

Despite the intriguing findings made in cell culture, the role of TMPRSS2 and HAT in influenza virus and SARS-CoV spread and pathogenesis remains to be defined. For this, it is essential to determine the extent of TMPRSS2 and HAT expression in viral target cells in human tissues. Here, we show that TMPRSS2 and HAT are coexpressed with ACE2 and 2,6-linked sialic acids, the key receptor determinants of SARS-CoV and influenza virus, respectively, in major portions of the human respiratory tract, indicating that these proteases could support SARS-CoV and influenza virus spread in humans. In addition, we demonstrate that HA activation is conserved between human TMPRSS2 and TMPRSS2 of animal species critically involved in zoonotic transmission of influenza virus, underlining a potentially important role of this protease in the influenza virus zoonosis.

## Materials and Methods

### Cell culture

293T cells were obtained from the American Type Culture Collection (ATCC) and were propagated in Dulbecco's modified Eagle's medium (DMEM) supplemented with 10% fetal bovine serum (FBS), penicillin and streptomycin, and grown in a humidified atmosphere of 5% CO_2_.

### Cell-cell fusion assay

For analysis of cell-cell fusion, 293T effector cells seeded in 6-well plates were CaPO_4_-transfected with either empty pcDNA plasmids or plasmids encoding SARS- S in combination with plasmid pGAL4-VP16 encoding the Herpes Simplex transactivator VP16 fused to GAL4, as described [13]. In parallel, 293T target cells were seeded in 48-well plates and transfected with plasmids encoding the indicated proteases or empty plasmid together with plasmid pGal5-luc, which encodes a promoter with five Gal4 binding sites in front of a luciferase gene. Transfected effector and target cells were mixed, incubated with trypsin or PBS and fusion was quantified by determination of luciferase activities in cell lysates 48 h after cocultivation using a commercially available kit (Promega, Madison, USA).

### Production of lentiviral pseudotypes and infection experiments

For generation of lentiviral pseudoparticles, CaPO_4_ transfections were performed as described [13]. Briefly, 293T cells were transiently cotransfected with pNL4-3 E-R- Luc [16] and expression plasmids coding for influenza virus HA and neuraminidase (NA) or vesicular stomatitis virus glycoprotein (VSV-G) [10]. For analysis of HA activation by TTSPs, expression plasmids for the indicated proteases [11], [13] or empty vector were cotransfected into cells producing pseudoparticles. The culture medium was replaced at 16 h and harvested at 48 h post transfection. The supernatants were passed through 0.45 µm filters and stored at −80°C. For infection, pseudoparticles were treated with either PBS or trypsin followed by incubation with 293T target cells for three days before cells were lysed and luciferase-activities determined using a commercially available kit (Promega, Madison, USA).

### Analysis of SARS-S and 1918 HA cleavage

For the detection of HA and SARS-S-cleavage in cis, 293T cells were cotransfected with plasmids encoding SARS-S [17] or 1918 HA and plasmids encoding the indicated proteases or empty vector (pcDNA). For analysis of SARS-S cleavage in trans, plasmids encoding SARS-S [17] and proteases were transfected separately into 293T cells followed by mixing of the transfected cells. Subsequently, the cells were treated with PBS or trypsin, lysed, separated via 12,5% SDS-PAGE and transferred onto nitrocellulose membranes. SARS-S was detected by staining with rabbit serum raised against the S1 subunit of SARS-S subunit [18]. For detection of HA, a mouse monoclonal antibody was used [19]. As a loading control, the stripped membranes were incubated with an anti-ß-actin antibody (Sigma, Deisenhofen, Germany). Bound antibodies were detected with HRP-coupled secondary antibodies (Dianova, Hamburg, Germany).

### Immunostaining of tissue sections

Formalin fixed paraffin embedded tissue samples of a wide range of tissues from the respiratory and gastrointestinal tracts, as well as the myocardium, were obtained from the Oxford Radcliffe Biobank, with full ethical approval from the National Research and Ethics Service (Oxfordshire Research and Ethics Committee A: reference 04/Q1604/21). While all patients gave generic consent for the use of their tissue in research at the time of signing a consent form for surgery, informed consent from each patient for the use of tissue in this study was not required by the National Research and Ethics Service, because all tissue was anonymised. Tissue sections were immunostained for TMPRSS2, HAT and ACE2 or with the elderberry lectin, *Sambucus nigra*, that detects 2,6-linked sialic acids. Antigen retrieval was performed by pressure cooking in different antigen retrieval solutions. Slides were mounted in Aquatex mounting medium (Merck, UK). ACE2 immunostaining (affinity purified goat polyclonal serum, R&D Systems, Abingdon, UK) was performed and detected using a mouse anti-goat Ig (GTI-75) [20] and the Novolink™ max polymer detection system (Leica Microsystems, Newcastle, UK), as per the manufacturer's instructions after antigen retrieval in citrate pH 6.0. TMPRSS2 (mouse monoclonal antibody P5H9 A3 ascites, a generous gift from Dr J.M. Lucas, Division of Human Biology, Fred Hutchinson Cancer Research Center, Seattle, WA 98109, USA, [21]) immunostaining was detected using the NovolinkTM max polymer detection system after antigen retrieval in Tris EDTA pH9.0. HAT immunostaining (mouse monoclonal antibody 337029, R&D Systems, Abingdon, UK) was performed using the Novolink™ max polymer detection system after antigen retrieval in Dako Target retrieval solution pH 6.0 (Dako, Cambridge, UK). Biotinylated elderberry lectin (Sambucus nigra) (Vector labs, Peterborough, UK) binding was detected using Streptavidin-HRP (Ar-Med Limited, Egham, UK) and detected using Dako DAB chromogen substrate (Dako, Cambridge, UK) after antigen retrieval in Tris-EDTA pH 9.0. Lung tissue was used as a positive control for TMPRSS2, ACE2 and elderberry lectin staining [11], [13], while bronchus was used as a positive control for HAT immunostaining [22]. As a negative control for ACE2 immunostaining and elderberry lectin staining, normal goat polyclonal serum was substituted for the primary antibody/lectin staining step. As a negative control for TMPRSS2 and HAT immunostaining, an irrelevant mouse monoclonal (anti-ALK1 antibody, clone ALK1 [23]) was substituted for the primary antibody. Stained sections were photographed with a Nikon DS-FI1 camera with a Nikon DS-L2 control unit (Nikon UK Limited, Kingston-upon-Thames, UK) and an Olympus BX40 microscope (Olympus UK Limited, Watford, UK).

## Results

Influenza viruses circulate in birds and poultry, with water fowl constituting the natural reservoir, and coinfection of swine with different influenza viruses is believed to play an important role in the emergence of pandemic viruses [1], [3]. TMPRSS2, TMPRSS4 and HAT were shown to activate influenza virus in transfected cells [4], [9], [10], but only for TMPRSS2 further evidence for a potential contribution to viral spread in humans was reported [11], [12]. In order to assess the role of TMPRSS2 in the influenza virus zoonosis, we tested whether this protease derived from chicken and swine is able to cleave HA. In parallel, we examined if TMPRSS2 of mouse origin facilitates HA proteolysis, since mice are commonly used as a model system for influenza virus spread and pathogenesis. Finally, cleavage and activation of HA by human and mouse HAT and TMPRSS4 were also evaluated.

**Figure 1:**
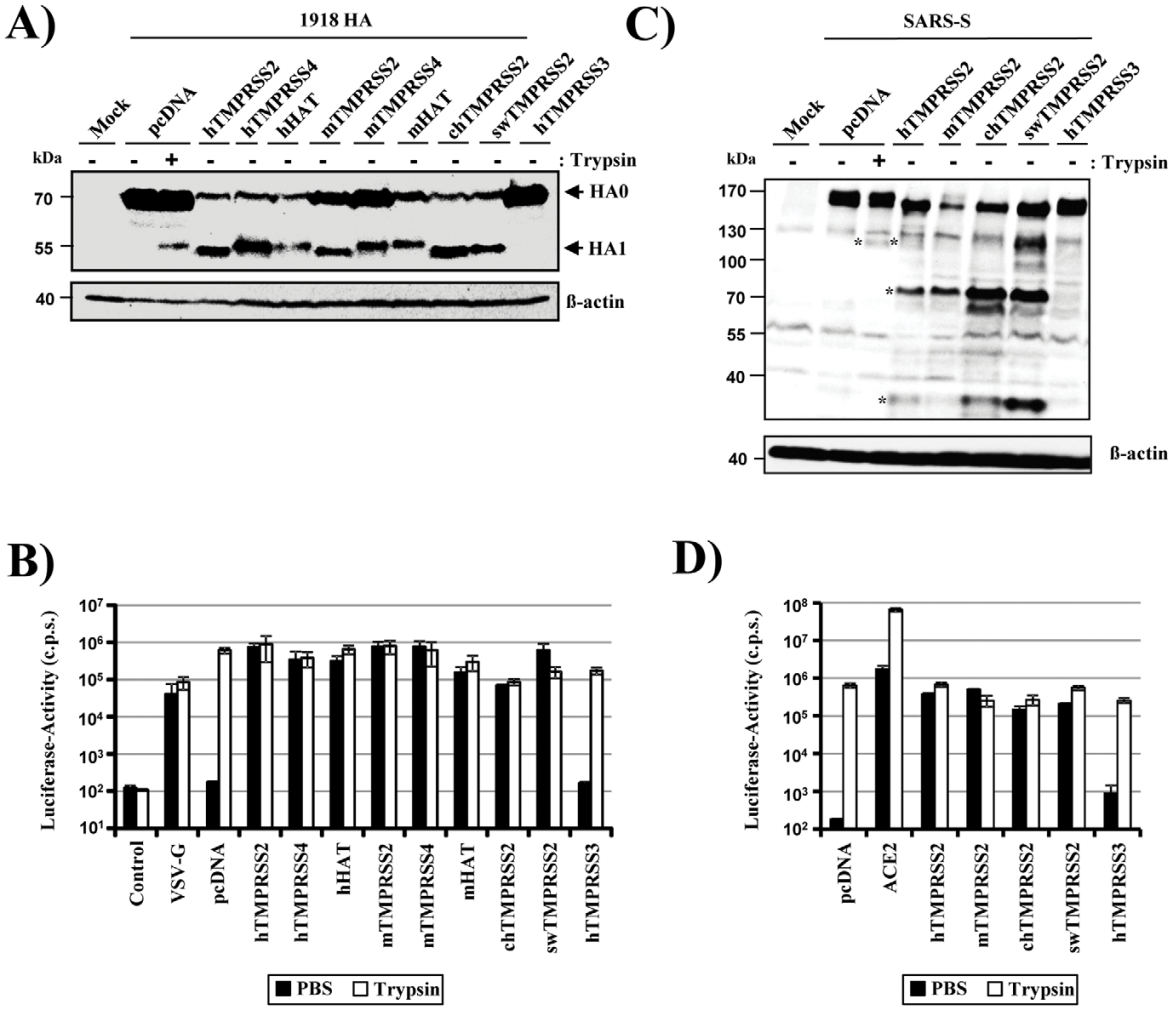
Proteolytic activation of influenza virus hemagglutinin and SARS spike protein is conserved between TMPRSS2 of human, porcine, avian and murine origin. (A) Expression plasmids encoding the HA of the 1918 influenza virus and the indicated proteases or empty vector (pcDNA) were transiently cotransfected into 293T cells. The cells were then treated with PBS or trypsin, and HA cleavage was detected by Western blot analysis of cell lysates using a monoclonal antibody specific for HA. Detection of ß-actin served as loading control. (B) Lentiviral reporter viruses bearing 1918 HA and NA or the VSV-G glycoproteins were generated in 293T cells coexpressing the indicated proteases or empty vector (pcDNA), treated with PBS (black bars) or trypsin (white bars), and used for infection of 293T target cells. Viruses harboring no glycoprotein were generated in parallel as control. Luciferase activities in the cell lysates were determined at 72 h post infection. The results of a representative experiment performed in triplicates are shown. Error bars indicate standard deviation (SD). Comparable results were obtained in a separate experiment. (C) To detect SARS-S cleavage in cis, expression plasmids coding for SARS-S and the indicated proteases or empty vector (pcDNA) were transiently cotransfected into 293T cells, which were then treated with trypsin or PBS. Subsequently, S-protein cleavage was detected by Western blot analysis of cell lysates using a serum specific for the S1 subunit of SARS-S. SARS-S cleavage fragments produced by trypsin and TMPRSS2 are indicated by asterisks. Detection of ß-actin served as a loading control. (D) Effector 293T cells were cotransfected with a SARS-S expression plasmid and a plasmid encoding GAL4-VP16 and mixed with target cells cotransfected with a plasmid encoding a GAL4-VP16 responsive luciferase expression cassette and an ACE2 expression plasmid or protease expression plasmid or empty plasmid. The effector and target cells were mixed, treated with PBS (black bars) or trypsin (white bars) and the luciferase activities in cell lysates quantified at 48 h after cell mixing. The results of a representative experiment performed in triplicates are shown. Error bars indicate standard deviation (SD). Similar results were observed in two independent experiments.

Western blot analysis of transfected cells revealed that TMPRSS2, TMPRSS4 and HAT of all animal species tested cleaved the HA precursor HA0 and produced HA1 cleavage fragments identical to those observed for the human enzymes (Fig. 1A). The slightly faster migration of HA1 fragments generated by TMPRSS2 compared to the other proteases is due to differential HA glycosylation [11]. Cleavage resulted in HA activation, since lentiviral vectors produced in the protease expressing cells were fully infectious in the absence of trypsin treatment (Fig. 1B). In contrast, no HA cleavage and activation was observed in cells transfected with empty vector or cells expressing human TMPRSS3 (Fig. 1A,B), which was previously demonstrated not to process HA [11]. Similarly, swine, chicken and mouse TMPRSS2 cleaved SARS-S into multiple fragments, as previously documented for human TMPRSS2 [13], although some variation in cleavage efficiency was noted (Fig. 1C). In contrast, trypsin digestion produced the S1 subunit, as expected [24], [25]. Of note, SARS-S was cleaved by TMPRSS2 upon coexpression of both proteins (cis cleavage, Figure 1C) and upon mixing of SARS-S expressing cells with protease expressing cells (trans cleavage, Fig. S1), although some variability in cleavage efficiency was noted in the latter setting. In agreement with SARS-S trans cleavage, expression of TMPRSS2 in target cells (TMPRSS2 panels) endogenously expressing very low amounts of viral receptor, ACE2 [5], allowed efficient SARS-S-driven cell-cell fusion and fusion efficiency was not increased by the addition of trypsin. In contrast, SARS-S-driven fusion with control transfected cells (pcDNA panel) was inefficient and fusion efficiency was rescued by trypsin treatment (Fig. 1D). Finally, transfection of ACE2 plasmid into target cells (ACE2 panel) also boosted cell-cell fusion and fusion efficiency was only modestly increased by trypsin, in agreement with our previous finding that receptor and protease expression on target cells can both limit SARS-S-mediated cell-cell fusion [26]. In sum, these results demonstrate that cleavage-activation of influenza HA and SARS-S is conserved between human, porcine, avian and murine TMPRSS2 as well as human and murine HAT. Our observations also suggest that TMPRSS2 can support influenza virus spread in species integral to the influenza zoonosis, and that mice are suitable models to study the role of TMPRSS2, TMPRSS4 and HAT in viral spread and pathogenesis.

**Figure 2:**
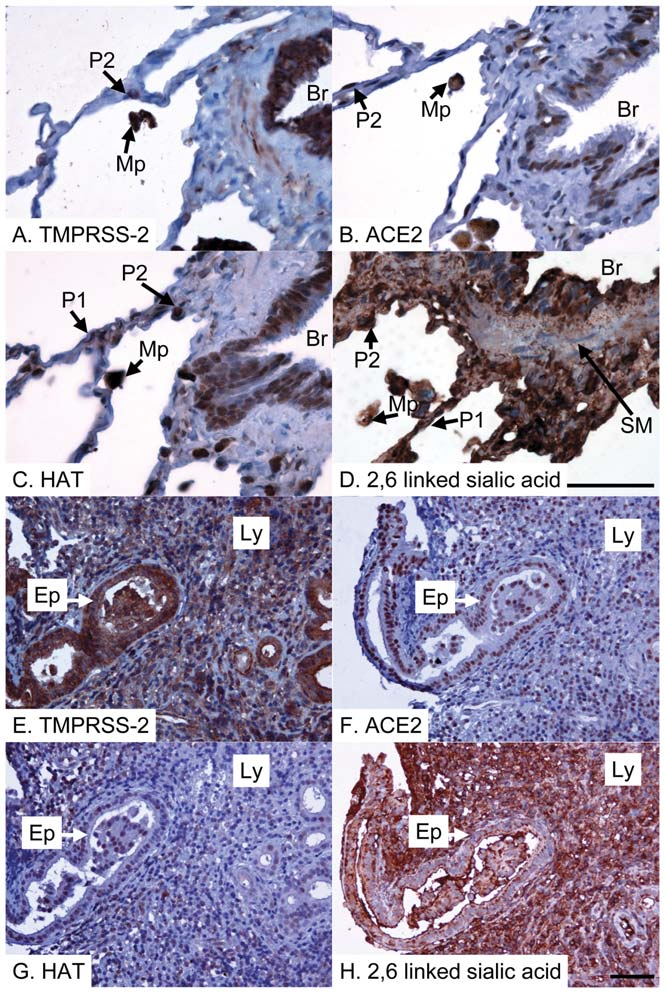
Pulmonary and respiratory sinus expression of SARS-CoV and influenza virus activating proteases and receptors. Lung (A–D) and sinus (E–H) tissue immunostained for TMPRSS2 (A&E), ACE2 (B&F) and HAT (C&G), or stained for 2,6-linked sialic acid (D&H; detected with elderberry (*Sambucus nigra*) lectin). All positive reactions are detected with the peroxidase technique (brown) and the tissue is counterstained with haematoxylin (blue). (A) There is strong positive anti-TMPRSS2 immunostaining of bronchial epithelium (lining the bronchus, marked Br), type 2 pneumocytes (P2) and alveolar macrophages (Mp). (B) There is moderately strong positive anti-ACE2 immunostaining of bronchial epithelium (lining the bronchus, marked Br), type 2 pneumocytes (P2) and alveolar macrophages (Mp). (C) There is moderately positive anti-HAT immunostaining of bronchial epithelium (lining the bronchus, marked Br) and alveolar macrophages (Mp), with weakly positive immunostaining of some type 1 (P1) and type 2 pneumocytes (P2). (D) All structures are strongly stained for 2,6-sialic acid except for smooth muscle (SM). (E) There is strong positive anti-TMPRSS2 immunostaining of sinus epithelium (Ep) and lymphoid cells (Ly). (F) There is strong positive anti-ACE2 immunostaining of sinus epithelium (Ep) and lymphoid cells (Ly). (G) There is moderately strong anti-HAT immunostaining of sinus epithelium (Ep) and occasional weakly positive immunostaining of lymphoid cells (Ly). (H) All structures are strongly stained for 2,6-sialic acid. Scale bar = 50 microns (shown in panels D and H and also pertaining to 3 preceding panels in each case).

**Figure 3:**
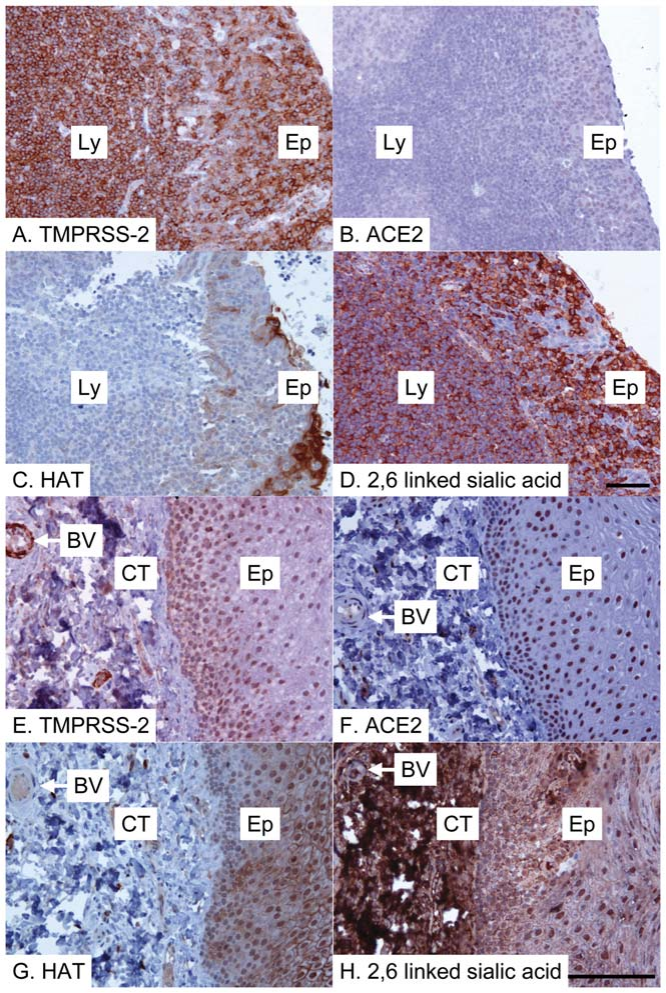
Tonsil and buccal mucosal expression of SARS-CoV and influenza virus activating proteases and receptors. Tonsil (A–D) and buccal mucosa (E–H) immunostained for TMPRSS2 (A&E), ACE2 (B&F) and HAT (C&G), or stained for 2,6-linked sialic acid (D&H; detected with elderberry (*Sambucus nigra*) lectin). All positive reactions are detected with the peroxidase technique (brown) and the tissue is counterstained with haematoxylin (blue). (A) There is strong positive anti-TMPRSS2 immunostaining of tonsillar epithelium (Ep) and lymphocytes (Ly). (B) There is weakly positive anti-ACE2 immunostaining of tonsillar epithelium (Ep), but little obvious positive immunostaining of lymphocytes (Ly). (C) There is strongly positive anti-HAT immunostaining of the basal and superficial, but not the middle, layers of tonsillar epithelium (Ep), but little obvious positive immunostaining of lymphocytes (Ly). (D) All structures are strongly stained for 2,6-sialic acid except for a few areas of cells within the tonsillar epithelium (Ep). (E) There is strong positive anti-TMPRSS2 immunostaining of buccal epithelium (Ep) and of a blood vessel (BV) in the underlying connective tissue (CT). (F) There is strong positive anti-ACE2 immunostaining of buccal epithelium (Ep) and weaker positive immunostaining of a blood vessel (BV) in the underlying connective tissue (CT). (G) There is strong positive anti-HAT immunostaining of buccal epithelium (Ep), but a blood vessel (BV) in the underlying connective tissue (CT) appears negative. (H) All structures are strongly stained for 2,6-sialic acid. Scale bar = 50 microns (shown in panels D and H and also pertaining to 3 preceding panels in each case).

**Figure 4:**
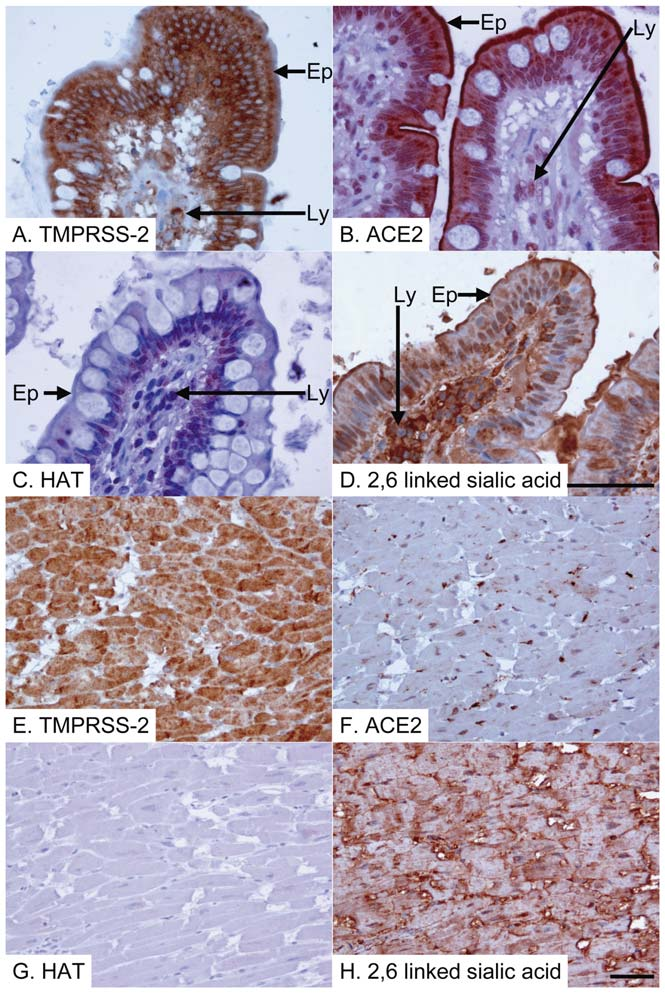
Ileal & myocardial expression of SARS-CoV and influenza virus activating proteases and receptors. Ileum (A–D) and myocardium (E–H) immunostained for TMPRSS2 (A&E), ACE2 (B&F) and HAT (C&G), or stained for 2,6-linked sialic acid (D&H; detected with elderberry (*Sambucus nigra*) lectin). All positive reactions are detected with the peroxidase technique (brown) and the tissue is counterstained with haematoxylin (blue). (A) There is strong positive anti-TMPRSS2 immunostaining of ileal epithelium (Ep) and also of lymphocytes (Ly) within the core of the villus. (B) There is strong positive anti-ACE2 immunostaining of ileal epithelium (Ep) and also of lymphocytes (Ly) within the core of the villus. (C) There is strongly positive anti-HAT immunostaining of the basal part of the ileal epithelial cells (Ep), but only weak positive immunostaining of occasional lymphocytes (Ly) within the villus core. (D) All structures are strongly stained for 2,6-sialic acid, including ileal epithelium (Ep) and lymphocytes (Ly). (E) There is strong positive anti-TMPRSS2 immunostaining of cardiac myocytes. (F) There is strong positive anti-ACE2 immunostaining of some cardiac myocytes. (G) There is no anti-HAT immunostaining of cardiac myocytes. (H) There is strong 2,6-sialic acid staining of cardiac myocytes. Scale bar = 50 microns (shown in panels D and H and also pertaining to 3 preceding panels in each case).

Binding of human influenza viruses to 2,6-linked sialic acids present on proteins and lipids on the host cell surface is critical for infectious viral entry into host cells [6]. We assessed whether TMPRSS2 and HAT are coexpressed with 2,6-linked sialic acid human tissues. Immunostaining demonstrated the presence of 2,6-linked sialic acids on the surface of almost all cell types (Fig. 2, 3, 4), in keeping with previous results [27]–[30], with the notable exception of vascular smooth muscle cells (data not shown), suggesting that expression of proteases, such as TMPRSS2 and HAT, but not 2,6-linked sialic acid is likely to be a major determinant of viral tropism. TMPRSS2 was expressed by epithelial cells at all sites examined in the aerodigestive tracts, as well as by many endothelial cells and myocytes of blood vessels, leucocytes (including alveolar macrophages) and smooth muscle cells (Fig. 2, 3, 4, table 1), indicating that TMPRSS2 could activate influenza virus in most permissive epithelia. HAT showed a distribution similar to TMPRSS2 (Fig. 2, 3, 4, table 1), but immunostaining of pneumocytes (alveolar epithelial cells) was weaker, implying low levels of protease expression at this site (Fig. 2A, C). Unlike TMPRSS2, which appeared to be expressed by the majority of type 2 pneumocytes, HAT was expressed by fewer than 50% type 2 pneumocytes, but was additionally seen to be expressed by occasional type 1 pneumocytes (Fig. 2A, C). Type 2 pneumocytes are defined by their morphology rather than a particular immunophenotype, being plump rather than flattened epithelial cells [31]. All sections were examined by an experienced consultant pathologist (ES) in order to identify the cell types that were immunopositive. The exact intensities of staining for TMPRSS2 and HAT of various epithelial types in the aerodigestive tracts are summarized in table 1. While TMPRSS2 expression by bronchial and intestinal smooth muscle cells was noted, these cells appeared negative for HAT, although some vascular smooth muscle cells were found to be positive (table 1). Interestingly, TMPRSS2 but not HAT was expressed by cardiac myocytes (Fig. 4), suggesting that influenza myocarditis might be promoted by TMPRSS2 but not HAT. Notwithstanding, our data demonstrate the potential importance of both proteases in influenza infection.

**Table 1:** Expression pattern of influenza virus and SARS-coronavirus activating proteases and receptors in human tissues.

Antigen/site	Cells/structure	TMPRSS2	HAT	ACE2	2,6-linked sialic acid
***Lung***	*Alveolar Epithelium*	type 2 not type 1 pneumocytes+	occasional type 2 & type 1 pneumocytes weakly+	type 2 not type 1 pneumocytes+	type 2 & type 1 pneumocytes+
	*Bronchial epithelium*	+	+	+	+
	*Alveolar macrophages*	+	+	+	+
	*Other*		interstitial macrophages/dendritic cells+; bronchial smooth muscle cells weakly+	some interstitial macrophages/dendritic cells	+at all sites, except the majority of smooth muscle cells (SMC)
***Bronchus & Larynx***	*Epithelium*	+on respiratory, glandular, transitional and (weakly on) squamous epithelium	+on respiratory, transitional and squamous, but not glandular epithelium	weakly+on respiratory & transitional epithelium; strongly+on glandular epithelium	+on respiratory, glandular, transitional and squamous epithelium
***Trachea***	*Epithelium*	+on respiratory, glandular, transitional and (weakly on) squamous epithelium	+on respiratory, transitional and squamous, but not glandular epithelium	−	+on respiratory, glandular, transitional and squamous epithelium
***Vocal folds & epiglottis***	*Squamous epithelium*	weakly+	+	−	+
***Buccal mucosa***	*Squamous epithelium*	+	+	+	+
***Nasal mucosa & respiratory sinuses***	*Epithelium*	+	+respiratory & transitional epithelium	+respiratory, transitional & glandular epithelium	+
	*Lymphocytes*	+	Occasionally+	Variably+	−
***Tonsil***	*Squamous epithelium*	+	+	weakly+	+
	*Lymphocytes*	+	−	−	+
***Oesophagus***	*Squamous epithelium*	weakly+	+	weakly+	+
***Stomach***	*Epithelium*	+	+	weakly+	+
***Ileum***	*Epithelium*	+	weakly+	+	+
***Colon***	*Epithelium*	+	weakly+	+	+
***Myocardium***	*Myocytes*	+	−	+	+
***Blood vessels***	*Endothelial cells*	some weakly+	variably+	variably+	all+
	*Vascular smooth muscle cells*	*+*	variably+	variably+	*−*
***Leucocytes***	*Lymphocytes*	+	weakly+(negative in tonsil)	+(negative in tonsil)	+
	*Other leucocytes*	variably+	variably+	variably+	generally+
***Smooth muscle cells***	*Oesophagus, stomach, intestine, bronchus*	+	−	+	−

Cell types expressing TMPRSS2, ACE2, HAT, 2,6-linked sialic acid are marked+, while those that do not express these molecules are marked−. “weakly” refers to a low level of staining.

TMPRSS2 on target cells activates SARS-S on adjacent cells for cell-cell fusion and activates virion-associated SARS-S for infectious host cell entry [13]–[15]. A wide range of sites demonstrated coexpression of ACE2 and TMPRSS2, and could thus support SARS-CoV spread (table 1). Specifically, in the lung type 2, but not type 1 pneumocytes express both molecules, as do alveolar macrophages and the epithelial cells of intrapulmonary bronchi (Fig. 2A, B). In the upper respiratory tract, the epithelium of the bronchi, larynx, nasal mucosa and respiratory sinuses (Fig. 2 E, F) expresses both molecules, while ACE2 expression is absent from the trachea, vocal folds and epiglottis, although a previous study by Ren et al demonstrated ACE2 expression on the surface epithelium and mucus gland epithelium of trachea, similar to our findings in the larynx and bronchus [32]. This suggests that ACE2 expression may be variable but widespread in the upper airway. The epithelia of the tonsil (Fig. 3A, B) and buccal mucosa (Fig. 2E, F) express both TMPRSS2 and ACE2. Additionally, ACE2 expression by some interstitial macrophages/dendritic cells in intra-alveolar septa of the lung, adjacent to TMPRSS2-expressing type 2 pneumocytes. In the gastrointestinal tract, epithelial co-expression of TMPRSS2 and ACE2 was identified at all sites examined, namely the oesophagus, stomach, ileum and colon. The two molecules were also expressed in cardiac myocytes. Furthermore, variable expression of both molecules by endothelial cells and myoctes of blood vessels, leucocytes and smooth muscle cells was seen (Fig. 2, 3, 4, table 1). Taken together, these results suggest that TMPRSS2 could promote SARS-CoV spread in important target sites, the gastrointestinal and respiratory tracts (table 1).

## Discussion

Influenza virus and SARS-CoV hijack host cell proteases to acquire infectivity and for influenza it has been shown that broad spectrum protease inhibitors have therapeutic potential [33]–[35]. However, the proteases responsible for viral activation in the infected host are unclear, although several candidates have been suggested [7], [36]. Recent studies demonstrate that TMPRSS2 and HAT activate influenza virus [4], [9], [10] and SARS-coronavirus [13]–[15], [37] in cell culture. We show that both proteases are expressed on receptor-positive cells throughout most of the human respiratory tract and might thus support influenza virus and SARS-CoV spread in and between individuals. In addition, influenza virus activation was conserved between TMPRSS2 orthologues of human, porcine and avian origin, suggesting that zoonotically transmitted influenza viruses may engage TMPRSS2 to facilitate their activation.

Influenza viruses usually replicate in the tracheao-bronchial epithelium [38]–[40]. Spread in these tissues might be supported by both TMPRSS2 and HAT, which we found to be expressed by cells positive for 2,6-linked sialic acid in the nasal and buccal mucosa as well as in the epithelium of trachea, bronchus and larynx. If infection is associated with pneumonia, a complication more frequently observed with pandemic compared to seasonal influenza viruses, viral spread to the alveolar epithelium is observed [38]. Type I pneumocytes have been suggested to be major targets of influenza virus in the alveoli [40] and were found to be positive for 2,6-linked sialic acid in this study. The protease responsible for HA activation in these cells remains to be defined, since TMPRSS2 was absent from this cell type and expression of HAT was infrequent and weak. However, other studies found that type II pneumocytes are preferentially infected [41] and these cells were identified as positive for 2,6-linked sialic acid, TMPRSS2 and occasionally for HAT within the present study. The presence of cells positive for 2,6-linked sialic acid, TMPRSS2 and/or HAT was not limited to the respiratory tract, in keeping with published findings which demonstrate TMPRSS2 expression in the epithelia of several organs [21], [42]–[45]. It is therefore tempting to speculate that TMPRSS2 and HAT might also support viral spread outside the lung and might thus contribute to complications associated with influenza infection, like gastrointestinal manifestations, myocarditis and encephalopathy [38]. In sum, TMPRSS2 and, with the exception of the alveolar epithelium, HAT could activate influenza viruses throughout the respiratory tract and might support viral spread in extra-respiratory tissues.

The mode of cleavage activation is a major virulence determinant of avian influenza viruses [11], [46]–[48]. Viruses with a multi-basic cleavage site in HA are believed to be activated by ubiquitously expressed host cell proteases and can thus replicate systemically and cause severe disease [11]. In contrast, it has been posited that replication of viruses with a mono-basic cleavage site is confined to the aerodigestive tract, because the expression of as yet unidentified HA-activating protease(s) is limited to this organ [11]. Our results suggest that TMPRSS2 could be the elusive protease, but it remains to be demonstrated whether TMPRSS2 expression in waterfowl and poultry is indeed specific for the aerodigestive tract. HA activation was conserved between avian, porcine and human TMPRSS2, which share high sequence identity (see figure S2), indicating that TMPRSS2 might support influenza virus spread not only in the reservoir (waterfowl) and humans but also in an important intermediate host (swine).

The SARS-CoV causes a severe respiratory illness with fatal outcome in about 10% of the afflicted individuals. The metalloprotease ACE2 has been identified as the SARS-CoV receptor and expression of ACE2 on type II pneumocytes, the major viral target cells, and other pulmonary cells has been demonstrated [49]–[52]. A cornerstone study by Simmons and colleagues showed that infectious SARS-CoV entry into cell lines depends on the activity of endosomal cathepins, particularly cathepsin L [8], suggesting that cathepsin activity might be required for viral spread in the respiratory tract. However, several studies showed that TMPRSS2 activates SARS-CoV for cathepsin-independent host cell entry and demonstrated TMPRSS2 expression in type II pneumocytes [13]–[15]. In addition, a recent report indicates that HAT can promote SARS-S-driven cell-cell but not virus-cell fusion [37]. Thus, the SARS-CoV may use TTSPs in addition to or instead of cathepsins to ensure its activation in key target cells. Our analysis confirms and extends these findings by demonstrating that ACE2 and TMPRSS2 are coexpressed by cells in the nasal and buccal mucosa as well as in the epithelia of bronchus and larynx. Thus, one can speculate that, in the context of the infected host, cathepsin activity might not be essential for SARS-CoV spread in most parts of the respiratory tract. Experiments with cathepsin inhibitors and knock-out mice are required to address this possibility. The gastrointestinal tract is a well-established target of SARS-CoV [49] and it has actually been suggested that a wide range of tissues and organs can be infected by the virus [53]. Our finding that TMPRSS2 and ACE2 are coexpressed in colon and various other tissues indicates that also the extrarespiratory spread of SARS-CoV might be promoted by TMPRSS2.

Collectively, our findings are compatible with an important role of TMPRSS2 and HAT in influenza virus and of TMPRSS2 in SARS-coronavirus infection. Knock down of these proteases in primary pulmonary cells and the analysis of *Tmprss2* knock-out mice would allow to precise definition of the role of these proteases in viral spread. The latter animals are available [54] and do not show an obvious phenotype, indicating that TMPRSS2 might be an attractive target for novel drugs active against respiratory viruses.

## Supporting Information

Figure S1 **To detect SARS-S cleavage in trans, expression plasmids coding for SARS-S and the indicated proteases or empty vector (pcDNA) were separately transfected into 293T cells.** Subsequently, the SARS-S and protease expressing cells were mixed, treated with trypsin or PBS and S-protein cleavage was detected by Western blot analysis using a serum specific for the S1 subunit of SARS-S. SARS-S cleavage fragments produced by trypsin and TMPRSS2 are indicated by asterisks. Detection of ß-actin served as a loading control.
(TIF)

Figure S2 **The amino acid sequences of TMPRSS2 of human (NCBI Reference SequenceNP_001128571.1), chicken (XM_416737.3), swine (BAF76737.1) and mouse origin (AAF97867.1) were aligned using VectorNTI. Identical amino acids are marked in black, similar amino acids are marked in grey.**
(TIF)
