# Supplementary figures and images for: Influenza and SARS-Coronavirus Activating Proteases TMPRSS2 and HAT Are Expressed at Multiple Sites in Human Respiratory and Gastrointestinal Tracts
Source: PLoS One. 2012 Apr 30;7(4):e35876. doi: 10.1371/journal.pone.0035876 (PMC3340400; doi:10.1371/journal.pone.0035876)

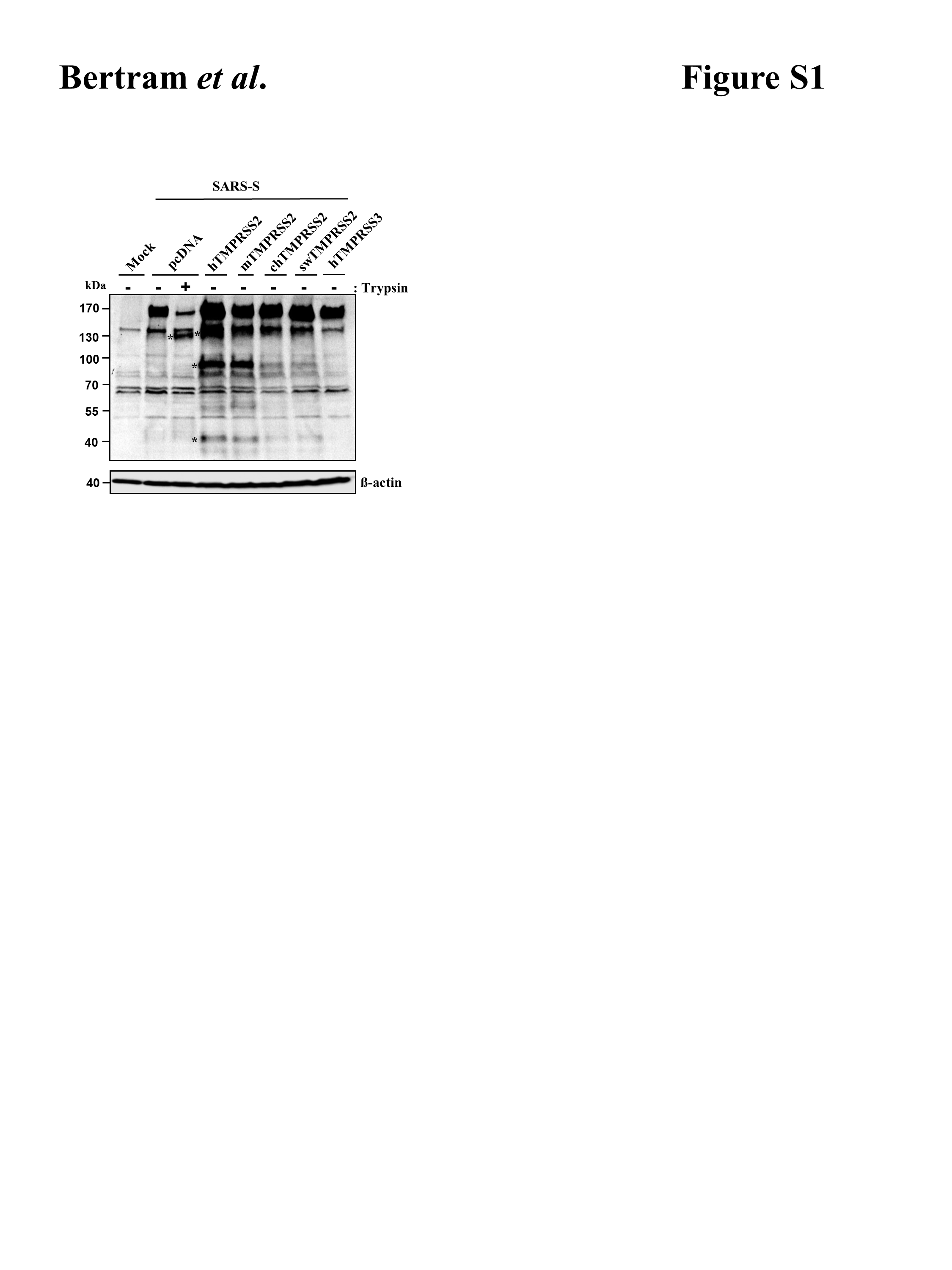

Supplement: Figure S1 — To detect SARS-S cleavage in trans, expression plasmids coding for SARS-S and the indicated proteases or empty vector (pcDNA) were separately transfected into 293T cells. Subsequently, the SARS-S and protease expressing cells were mixed, treated with trypsin or PBS and S-protein cleavage was detected by Western blot analysis using a serum specific for the S1 subunit of SARS-S. SARS-S cleavage fragments produced by trypsin and TMPRSS2 are indicated by asterisks. Detection of ß-actin served as a loading control. (TIF) [file pone.0035876.s001.tif]

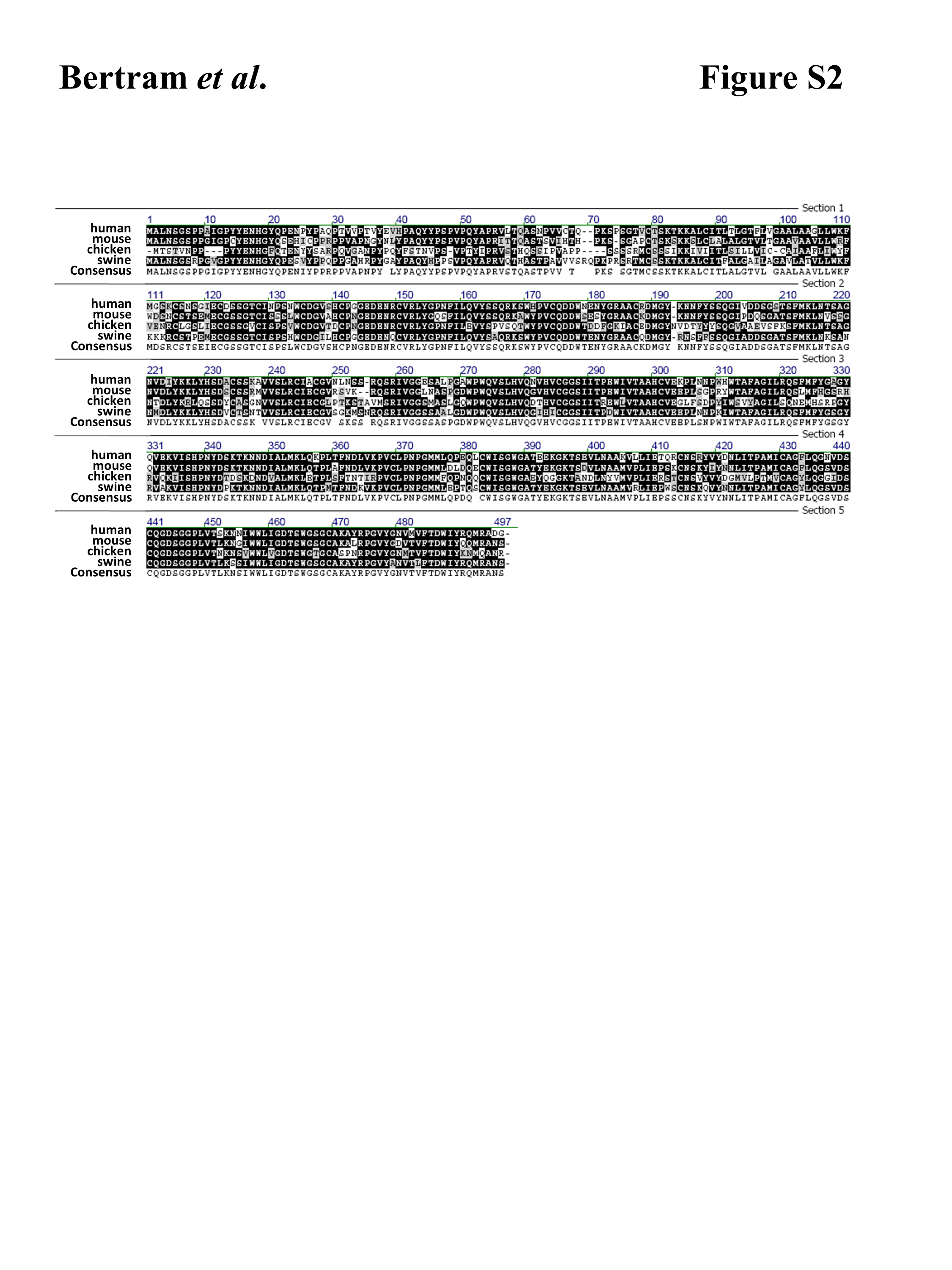

Supplement: Figure S2 — The amino acid sequences of TMPRSS2 of human (NCBI Reference SequenceNP_001128571.1), chicken (XM_416737.3), swine (BAF76737.1) and mouse origin (AAF97867.1) were aligned using VectorNTI. Identical amino acids are marked in black, similar amino acids are marked in grey. (TIF) [file pone.0035876.s002.tif]
